# Density functional theory study the effects of oxygen-containing functional groups on oxygen molecules and oxygen atoms adsorbed on carbonaceous materials

**DOI:** 10.1371/journal.pone.0173864

**Published:** 2017-03-16

**Authors:** Xuejun Qi, Wenwu Song, Jianwei Shi

**Affiliations:** 1 School of Architecture and Civil Engineering, Xihua University, Chengdu, Sichuan, China; 2 School of Energy and Power Engineering, Xihua University Chengdu,Sichuan, China; SPECS Surface Nano Analysis GmbH, GERMANY

## Abstract

Density functional theory was used to study the effects of different types of oxygen-containing functional groups on the adsorption of oxygen molecules and single active oxygen atoms on carbonaceous materials. During gasification or combustion reactions of carbonaceous materials, oxygen-containing functional groups such as hydroxyl(-OH), carbonyl(-CO), quinone(-O), and carboxyl(-COOH) are often present on the edge of graphite and can affect graphite’s chemical properties. When oxygen-containing functional groups appear on a graphite surface, the oxygen molecules are strongly adsorbed onto the surface to form a four-member ring structure. At the same time, the O-O bond is greatly weakened and easily broken. The adsorption energy value indicates that the adsorption of oxygen molecules changes from physisorption to chemisorption for oxygen-containing functional groups on the edge of a graphite surface. In addition, our results indicate that the adsorption energy depends on the type of oxygen-containing functional group. When a single active oxygen atom is adsorbed on the bridge site of graphite, it gives rise to a stable epoxy structure. Epoxy can cause deformation of the graphite lattice due to the transition of graphite from sp^2^ to sp^3^ after the addition of an oxygen atom. For quinone group on the edge of graphite, oxygen atoms react with carbon atoms to form the precursor of CO_2_. Similarly, the single active oxygen atoms of carbonyl groups can interact with edge carbon atoms to form the precursor of CO_2_. The results show that oxygen-containing functional groups on graphite surfaces enhance the activity of graphite, which promotes adsorption on the graphite surface.

## Introduction

The chemical reaction between O_2_ and carbon is an important step in the process of coal gasification, the water-gas shift reaction, and other solid fuel gasification processes. Coal gasification is a promising strategy for more effective coal utilization because it releases less harmful gas emissions to the environment and it is more efficient than the direct combustion of coal. Recently, many theoretical studies have studied the oxidizing mechanism of carbonaceous materials to understand gas-solid reactions and changes to the graphite lattice[1–5]. Ghaderi and Peressi[6] employed density functional theory to investigate states of graphite that differed with respect to hydroxyl functional groups and found that hydroxyl is weakly adsorbed on a perfect graphite surface and strongly adsorbed on defected graphite. Atamny et al. [7] experimentally studied the interactions of oxygen molecules with the (0 0 1) face of highly-oriented pyrolytic graphite at 1000 K. Another group reported that chemical functional groups, such as hydroxyl, carbonyl, ether, epoxy and peroxy group often appear on the edge of graphite [8]. Ljubisa et al. [9, 10] reported that oxygen-containing functional groups can affect the migration of active oxygen atoms on the graphite surface and affect the value of the energy barrier. Espinal et al. [11] used a graphene model instead of carbonaceous materials and investigated how water molecules interact with the edge active sites of these materials. Sendt and co-workers [12–14] found that oxygen-containing functional groups or surface oxides appear on the graphite surface and can change the adsorption capacity of oxidized graphite and further influence subsequent reactions. In fact, many oxygen-containing functional groups can be present on the surface of carbon materials, such as carboxyl (-COOH), hydroxyl (-OH), and carbonyl (= CO) groups, as identified by X-ray photoelectron spectroscopy (XPS) and other methods [15–18]. The chemical properties of coal char can be significantly altered by the presence of these oxygen-containing functional groups. However, how oxygen-containing functional groups on the graphite surface affect the adsorption of oxygen molecules and other atoms remains unclear.

It is difficult to experimentally study the mechanism of oxygen molecules and oxygen atoms adsorbed on the graphite surface due to the multiple reaction steps that occur both in the gas phase and at the solid-gas interface. Instead, computational chemistry has been recently applied by many researchers to study the individual steps of the reactions of carbonaceous materials and small molecules [19–21]. In this study, density functional theory method was employed to investigate the effect of oxygen-containing functional groups on the adsorption of oxygen molecules and oxygen atoms on the graphite surface. Different oxygen-containing functional groups adsorbed on graphite surfaces were compared in reactions of oxygen atoms. The results of this study help to elucidate the role of oxygen-containing functional groups in the processes of carbonaceous material oxidation, gasification, and combustion.

## Computational details

**Fig 1:**
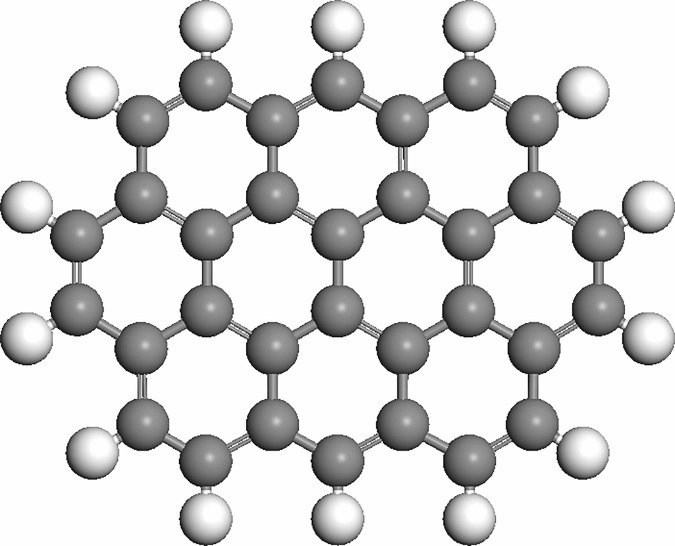
Graphite cluster model. (Carbon atoms shown in middle gray, and H atoms shown in light gray).

In general, carbonaceous materials are considered to act as macrostructures that are formed by aromatic clusters. The results of ^13^C NMR experiments indicate that char is connected by graphene clusters that consist of 12–25 aromatic carbon atoms [22], which is equivalent to 3–7 benzene rings per graphene unit. The reactivity of each graphene cluster is not influenced by the remaining char structure because electrons do not delocalize significantly through a single bond [23]. Chen and Yang found that the graphite model C_25_H_9_ was well-suited for producing graphite structures and the obtained parameters showed good agreement with experimental values [24]. Due to the negligible interactions between graphite layers, the graphite models used in theoretical studies typically consist of only a single graphite layer. The size of the graphite cluster model should ensure that oxygen atoms or oxygen molecules do not interact with the edge carbon atoms. This model has been successfully used in many studies of the adsorption of small molecules on graphite surfaces. In this study, a single graphene layer was used to represent a coal char model. The optimized geometric structure of the graphite cluster model is shown in Fig 1. Carbon atoms at the boundaries of the model were covered by H atoms to limit the domain size effect. In this model, the average length of C-C bonds is 0.1420 nm with dihedral angles of either 0° or 180°, indicating that this coal char model meets the calculation requirements.

Oxygen atoms can be bound to graphite models as components of different oxygen-containing functional groups. The edge sites are more reactive than the basal plane surface of graphite, resulting in the preferential adsorption of oxygen-containing functional groups on edge active sites. When oxygen-containing functional groups attach to edge sites, they can change the chemical adsorptive ability of graphite. In our model, the oxygen-containing functional groups = O, C = O, OH, and COOH were subject to pre-adsorption on the graphite edge, which simulates the oxidized surface. Density functional theory calculations were performed using DMol^3^ [25, 26]. The exchange-correlation energy was described with the Perdew and Wang scheme by using generalized gradient approximation (GGA), and Becke's corrected exchange functional was used in GGA calculations [27]. For the numerical basis sets, the double numerical plus polarization (DNP) was utilized because it can provide reasonable accuracy for energy calculation with efficient use of computational resources. In addition, van der Waals forces were also considered in the energy calculation. All orbitals including core electrons were considered for the process of computation. In order to ensure the accuracy of the calculations, the value of the tolerance self-consistent field (SCF) for geometry optimization was ≤1.0×10^−6^ and the convergence tolerance for energy was 2.0×10^-5^Ha.

Several steps were performed for the structural optimization of oxygen atoms and oxygen molecules interacting with graphite. First, the oxidized graphite was prepared and relaxed. Then, oxygen atoms or oxygen molecules were placed on three different adsorption sites: 1. at the center of the hexagons at 0.15 nm above the graphite plane (hollow sites); 2. in the middle point of a C-C bond at 0.15 nm above the graphite plane (bridge sites); and 3. on the top site of the carbon atom at 0.15 nm above the graphite plane (top sites). The different initial orientations of the oxygen atoms adsorbed on the surface were determined based on the lowest energy.

In this study, adsorption energy was defined according to the expression ΔE = E(substrate+X)-[E(X)+E(substrate)]. Here, E(X) is the energy of an isolated oxygen molecule or oxygen atom, E(substrate) is the energy at its optimized relaxed geometry in the absence of adsorption species, and E(substrate+X) corresponds to the energy at its optimized geometry structure upon adsorption of oxygen molecules or oxygen atoms on the graphite surface. Adsorption of the adsorbate is exothermic if ΔE is negative.

## Results and discussion

### Oxygen molecule adsorbed on graphite surface with different oxygen-containing functional groups

There are many recent studies of oxygen molecules adsorbed on the surface of graphite. Beran et al. [28] adopted quantum chemical methods to study the adsorption of oxygen molecules on graphite surfaces and found that oxygen molecules only physically adsorbed on the surface of graphite. Lamoen et al. [29] investigated the adsorption of oxygen molecules on clean surfaces of graphite, and found that the interactions between oxygen molecules and graphite are mutually repulsive. In contrast, Janiak and co-workers used experimental methods and reported that O_2_ molecules physisorb on a clean graphite surface at low temperature with 0.1 ev adsorption energy [30]. The previous studies both experimentally and theoretically consistently find that O_2_ molecules are weakly adsorbed on clean graphite basal surfaces.

**Fig 2:**
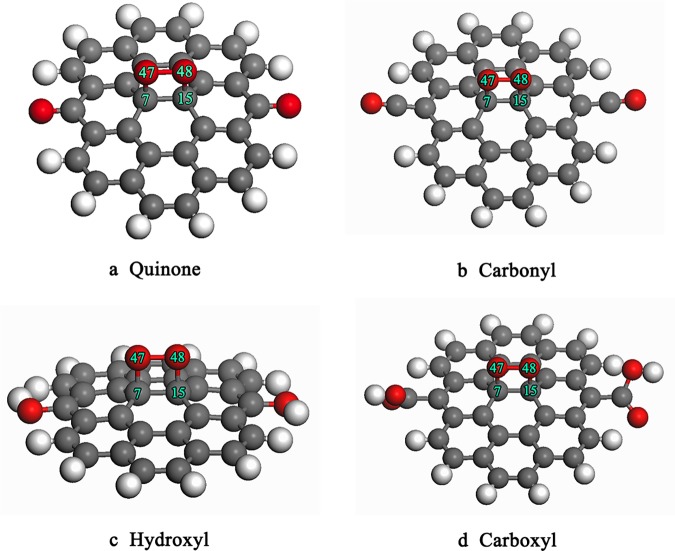
Optimized geometry structure of oxygen molecule adsorbed on the graphite withdifferent oxygen-containing functional groups. (Carbon atoms shown in middle gray, oxygen atoms shown in red and H atoms shown in light gray).

During the oxidization of carbonaceous materials, some fresh oxygen-containing functional groups frequently appear on the graphite edge, such as hydroxyl (-OH), carbonyl (-CO), quinone (-O), and carboxyl (-COOH) groups [15, 31]. These pre-adsorption, oxygen-containing functional groups alter the adsorption capacity of the carbonaceous material and may affect the adsorption of O_2_ molecules on the surface of graphite. For the convenience of research, four oxygen-containing functional groups (hydroxyl, carbonyl, quinone, and carboxyl groups) were placed symmetrically on the edge of cluster models to simulate a oxidized graphite surface. The final optimized configuration of oxygen molecules adsorbed on the oxidized graphite surfaces is shown in Fig 2. The main geometrical parameters and adsorption energy are listed in Table 1. The oxygen molecules were placed on different active sites of graphite, and the preferred orientation for the oxygen molecules adsorbed on graphite is parallel to C-C bonds. After the adsorption of an oxygen molecule, the oxygen atom interacts with the carbon atom to form four-member ring structure, in good agreement with previous research [32]. The formation of the C-O bond and the adsorption energy together indicate that O_2_ molecule chemisorbs on the oxidized graphite because oxygen-containing functional groups alter the chemical properties of graphite. It can be seen from Table 1 that the values of the adsorption energies are all positive, which indicates that the adsorption of oxygen molecules onto the oxidized graphite surface occurred via an endothermic reaction. In addition, the adsorption energy varied from 125.9 kJ/mol to 221.9 kJ/mol, suggesting that the adsorption energy depended on the type of local oxygen-containing functional groups.

**Table 1:** The main parameters of geometry structure and adsorption energy.

	C(7)-C(15)(nm)	C(7)-O(47)(nm)	C(15)-O(48)(nm)	O(47)-O(48)(nm)	ΔE(kJ/mol)
2a	0.1549	0.1495	0.1495	0.1493	147.8
2b	0.1550	0.1508	0.1508	0.1485	125.9
2c	0.1549	0.1507	0.1507	0.1497	218.1
2d	0.1555	0.1508	0.1508	0.1485	221.9

To explore the reasons for different adsorption energies, the Mulliken atomic charge distributions were analyzed. The changes in the atomic charges before and after the adsorption of oxygen molecules on the graphite surface are listed in Table 2. The results show that the charge of C(7) and C(15) were significantly different before and after the adsorption of oxygen molecules on graphite for the different oxygen-containing functional groups. The charge of an oxygen molecule adsorbed on the graphite is transferred between the oxygen atom and the graphite. Thus, electrons occupied the anti-bond orbitals of oxygen molecules, which decreased the bond energy of the oxygen molecule. This promotes the activation of the oxygen molecules and can enhance the reaction rate.

**Table 2:** Mulliken atomic charges.

		2a	2b	2c	2d
Before	C(7)	0.009	-0.004	0.005	0.014
	C(15)	0.009	-0.004	0.005	0.014
	C(7)	0.028	0.030	0.036	0.035
After	C(15)	0.028	0.030	0.036	0.035
	O(47)	-0.220	-0.233	-0.229	-0.224
	O(48)	-0.220	-0.233	-0.229	-0.224

### Oxygen atom adsorbed on the graphite surface

**Fig 3:**
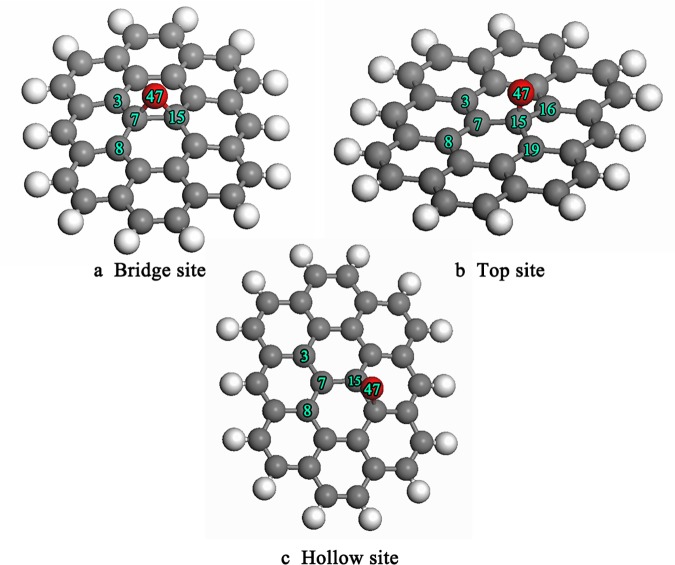
Final configuration of single oxygen atom adsorbed on the surface of graphite.

First, a single oxygen atom was placed on the bridge site of graphite with the final configuration as shown in Fig 3A. The oxygen atom adsorbed at the bridge site forms an epoxy structure, consistent with the previous experimental report [33]. The main parameters of the geometric structure and adsorption energy are listed in Table 3. As presented in the table, the bond length of C(7)-C(15) increased from 0.1420 nm to 0.1551 nm after adsorption of the oxygen atom on the bridge site. At the same time, the angle of C(8)-C(7)-C(15) changed from 120° to 117.6°. The observed change in bond length and bond angle indicates that there was a transfer of graphite from sp^2^ to sp^3^ hybridization. Next, the oxygen atom was placed on the top site, and the final configuration is shown in Fig 3B. The oxygen atom interacted with the carbon atom to form a C-O bond, and C(15) was pulled out of the basal plane by about 0.058 nm, which caused the neighboring bonds C(7)-C(15), C(7)-C(3), and C(7)-C(8) to increase to 0.1505 nm, 0.1503 nm, and 0.1504 nm. In the third test, the oxygen atom was placed on the hollow site, and the final configuration is shown in Fig 3C. The simulated results show that the oxygen atom directly interacted with the neighbor carbon atom to form an epoxy structure. Comparison of the adsorption energy values suggests that the adsorption energy is different for different adsorption sites. It is found that the oxygen atoms adsorbed more favorably on the top site and the bridge sites.

**Table 3:** The main parameters of geometry structure and adsorption energy.

	C(7)-C(15)(nm)	C(7)-C(8)(nm)	C(3)-C(7)(nm)	∠C(8)-C(7)-C(15)(°)	∠C(3)-C(7)-C(8)(°)	ΔE(kJ/mol)
3a	0.1551	0.1463	0.1463	117.6	119.1	-162.6
3b	0.1505	0.1504	0.1503	119.3	113.6	-122.2
3c	0.1469	0.1465	0.1532	119.9	117.8	-191.9

### Effect of different oxygen-containing functional groups on the adsorption of oxygen atoms on the graphite surface

**Fig 4:**
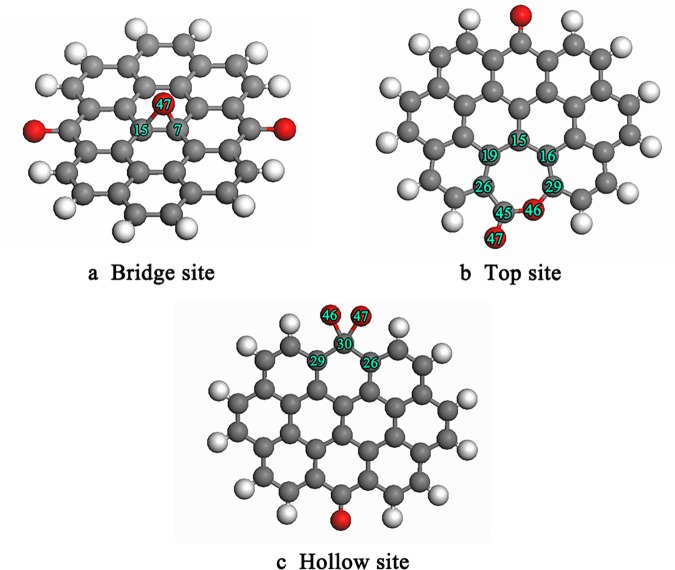
Final optimized geometry structure of single oxygen atom adsorbed on the graphite with quinone functional groups.

The effects of quinone functional groups on the adsorption of an oxygen atom on the graphite surface are presented in Fig 4. First, the single oxygen atom was placed on a bridge site, with the final optimized geometry structure as shown in Fig 4A. The oxygen atom combined with a carbon atom to form an epoxy structure. The bond length of C(7)-C(15) elongated from 0.1420 nm to 0.1506 nm after the adsorption of the oxygen atom. The bond order analysis shows that C(7)-C(15) decreased from 0.9995 to 0.5235. The change of bond length and bond order indicate that the C(7)-C(15) bond was weakened significantly. In the next test, the single atom was placed on a top site, with the optimized structure as shown in Fig 4B. The calculation results showed that the oxygen atom inserted into the C(29)-C(45) to form a seven-member ring structure. Next, the most likely pathway was the rupture of C(26)-C(45) and C(29)-O(46) bonds and the release of a CO_2_ molecule. Thus, the seven-member ring structure decomposed into a CO_2_ molecule and a five-member ring structure. In the last test, the single oxygen atom was placed on the top site and reached the final configuration depicted in Fig 4C. The oxygen atom directly combined with a carbon atom to form an O(47)-C(30)-O(46) structure. The Mulliken bond order of C(29)-C(30) and C(26)-C(30) decreased from 0.79 to 0.71, which means that C(29)-C(30) and C(26)-C(30) were activated after the introduction of the oxygen atom. In the next step, the possible reaction path may be the fracture of C(29)-C(30) and C(26)-C(30) bonds and the O(47)-C(30)-O(46) structure to leave the graphite surface in the form of CO_2_. This may be a path for the generation of CO_2_ during the gasification reaction.

**Fig 5:**
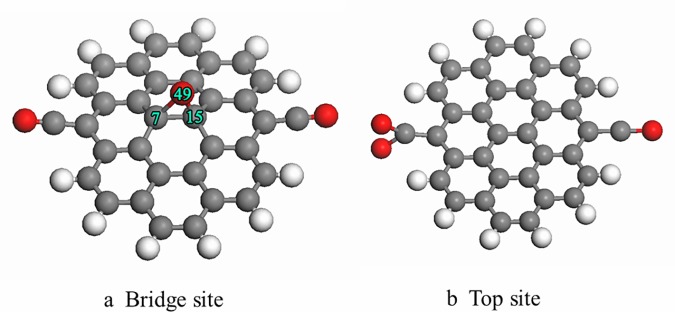
Final optimized geometry structure of oxygen atom adsorbed on the graphite with carbonyl functional groups.

Fig 5 shows the effect of carbonyl functional groups on oxygen atom adsorbed on the graphite surface. As occurred in the above simulations, when the single oxygen atom was placed on the bridge site, the oxygen atom interacted with a carbon atom to form an epoxy structure, as depicted in Fig 5A. The results indicated that the bond length of C(7)-C(15) increased from 0.1420 nm to 0.1493 nm, and the bond order reduced from 0.9995 to 0.5591. This suggests that C(7)-C(15) was greatly weakened due to the addition of the oxygen atom. Fig 5B shows the final structure formed when a single oxygen atom was placed on the top site of graphite. The oxygen atom interacted with carbonyl groups to form an O-C-O structure. This O-C-O structure is unstable, and the O-C-O structure was released from the basal plane in the form of gaseous CO_2_ due to the cleavage of the C-C bond. This may be the shortest path for the evolution of CO_2_ gas.

**Fig 6:**
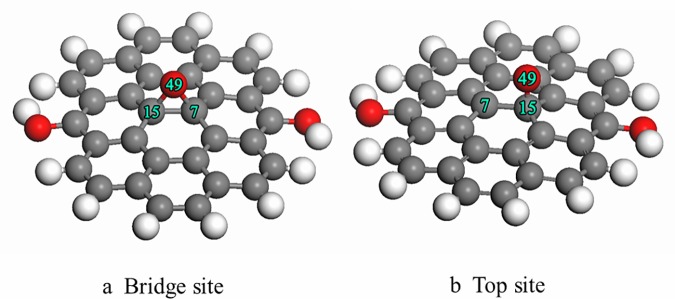
Final optimized geometry structure of single oxygen atom adsorbed on the graphite with hydroxyl functional groups.

Fig 6 presents the effect of hydroxyl functional groups on an oxygen atom adsorbed on the graphite surface. Fig 6A shows the final structure of a single oxygen atom adsorbed on the bridge site of graphite with hydroxyl functional groups. The bond length of C(7)-C(15) increased from 0.142 nm to 0.153 nm after the adsorption of the oxygen atom. Fig 6B shows the final structure of a single oxygen atom placed on the top site of graphite. The carbon atom underneath was pulled out from the basal plane, causing the deformation of the graphite surface. This may be due to the transition of carbon hybridization from sp^2^ to sp^3^.

**Fig 7:**
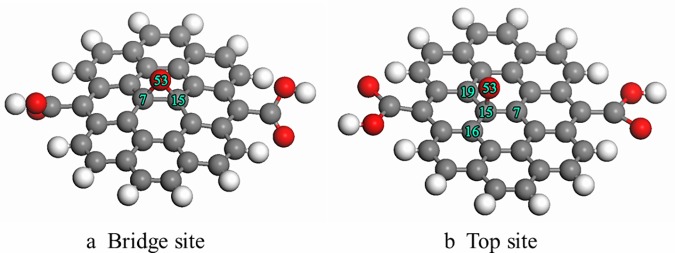
Final optimized geometry structure of single oxygen atom adsorbed on the graphite surface with carboxyl functional groups.

Carboxyl functional groups usually appear on an oxidized surface. Fig 7 shows the final optimized geometric structures of a single active oxygen atom adsorbed on the graphite surface with carboxyl functional groups. Fig 7A shows the final structure of a single oxygen atom adsorbed on the bridge site of graphite with carboxyl functional groups. The calculation results indicated that the bond length of C(7)-C(15) increased from 0.1420 nm to 0.1555 nm after adsorption of the oxygen atom on the bridge site. A single oxygen atom adsorbed on the top site to form a C-O bond, as demonstrated in Fig 7B. The results showed that the bond lengths of C(7)-C(15), C(15)-C(16), and C(15)-C(19) were 0.1505 nm, substantially longer than the 0.142 nm for the sp^2^ state.

The main parameters of geometric structure and the adsorption energy of an oxygen atom adsorbed on graphite with different oxygen-containing functional groups are summarized in Table 4. A similar geometry was observed for the final structures of a single oxygen atom adsorbed on the bridge site for the different graphite materias (Fig 4A, Fig 5A, Fig 6A and Fig 7A). However, these structures are significantly different in their released energy. The calculated adsorption energy for graphite with quinone functional groups and carbonyl functional groups were -238 kJ/mol and -253 kJ/mol, respectively. Thus, quinone functional groups and carbonyl functional groups on the graphite surface release more energy than the other two functional groups. To explore the reason for this phenomenon, the Mulliken atomic charges are listed in Table 5. The number of transfer charges differ for the different oxygen-containing functional groups and the transfer charge is closely related to the adsorption energy. Therefore, we conclude that oxygen-containing functional groups can influence charge transfer to further change the adsorption energy.

**Table 4:** Sumarize of the main parameters of geometry structure and adsorption energy.

	4a	5a	6a	7a
C(7)-C(15) (nm)	0.1506	0.1493	0.1526	0.1555
C(7)-O(47) (nm)	0.1460	-	-	-
C(7)-O(49) (nm)	-	0.1472	0.1461	-
C(7)-O(53) (nm)	-	-	-	0.1450
<C(7)-O(47)-C(15)(°)	62.1	-	-	-
<C(7)-O(49)-C(15)(°)	-	60.9	62.9	-
<C(7)-O(53)-C(15)(°)	-	-	-	64.8
ΔE (kJ/mol)	-238	-253	-168	-169

**Table 5:** Mulliken atomic charges.

	4a	5a	6a	7a
C(7)	0.096	0.092	0.102	0.107
C(15)	0.096	0.092	0.102	0.107
O(47)	-0.399	-	-	-
O(49)	-	-0.420	-0.412	-
O(53)	-	-	-	-0.398

## Conclusions

Density function theory combined with a graphite cluster model were used to investigate the adsorption of oxygen molecules and single oxygen atoms on a graphite surface with different oxygen-containing functional groups. The oxygen-containing functional groups altered the chemical properties of the graphite basal plane. When oxygen molecules were adsorbed on the basal plane, they formed a four-member ring structure and the O-O bond was greatly weakened. Additionally, the adsorption energy changed for the different types of oxygen-containing functional groups.

Single oxygen atoms that were adsorbed on the bridge sites of graphite formed epoxy structures. Graphite lattice deformation occurred due to the transition of graphite from sp^2^ to sp^3^ after the introduction of oxygen atoms. The calculation results suggest that adsorption reactions are exothermic processes. When quinone functional groups appeared on graphite, oxygen atoms directly interacted with carbon atom to form the precursor of CO_2_. For carbonyl functional groups adsorbed on the basal plane, the single oxygen atom interacted with the outer carbon atom to form the precursor of CO_2_. The presence of oxygen-containing functional groups on a graphite surface can attract more oxygen molecules and oxygen atoms for greater adsorption on the graphite surface.
